# Optical and UV-Aging Properties of LDH-Modified Bitumen

**DOI:** 10.3390/ma8074022

**Published:** 2015-07-03

**Authors:** Xing Liu, Shaopeng Wu, Gang Liu, Liping Li

**Affiliations:** State Key laboratory of Silicate Materials of Architectures, Wuhan University of Technology, Wuhan 430070, China; E-Mails: liuxing1107@whut.edu.cn (X.L.); wusp@whut.edu.cn (S.W.); lipingli@whut.edu.cn (L.L.)

**Keywords:** layered double hydroxides (LDHs), bitumen, optical, UV aging

## Abstract

Layered double hydroxides (LDHs) are an ultraviolet-light (UV) resistant material. In this study, LDHs were used to modify bitumen. The optical and UV aging properties of LDHs modified bitumen were investigated. Firstly, the thin films of bitumen, with and without LDHs, were prepared. By using the UV-Vis spectrophotometer, absorbance, reflectance, and transmittance of bituminous thin film were evaluated. The morphology of LDHs-modified bitumen was observed by using fluorescence microscopy (FM). Finally, the aging resistance of LDH-modified bitumen was investigated by using the UV-aging oven. Results indicated that the LDHs, especially with 5 wt % in the bitumen, can effectively absorb and reflect the UV light and improve the UV-aging resistance of bitumen. This implied that the addition of LDHs into bitumen had the potential to prolong the service life of asphalt pavement.

## 1. Introduction

Bitumen is used in the construction of asphalt pavement to function as a binder. The performance of bitumen can deteriorate due to aging [1,2]. There are two reasons for the aging of bitumen: one is thermo-oxidation during mixing, transportation and laying of asphalt mixtures; the other is photo-oxidation during the service life of the asphalt pavement [3,4,5]. The ultraviolet (UV) light plays a great role in the photo-oxidation of bitumen and makes the surface layer of asphalt pavement stiffer and brittle [4,6]. The UV light, a part of sunlight, has a shorter wavelength but with a higher energy, compared to visible and infrared light. According to the wavelength, UV light consists of three spectra: UV-A (320–400 nm), UV-B (290–320 nm), and UV-C (220–290 nm) [7]. UV-A can fracture the covalent bonds in the bitumen and is the main energy source to age the bitumen. Due to the blocking effect of the atmosphere, UV-B and UV-C hardly reach the surface of the Earth. Therefore, how to impede the UV light, especially UV-A, is very important to improving the aging resistance of the bitumen and prolong the service life of asphalt pavement. Many modifiers have been investigated to improve the aging resistance of the bitumen, like the organo-montmorillonite (OMMT), carbon black *etc*. [8,9].

**Figure 1:**
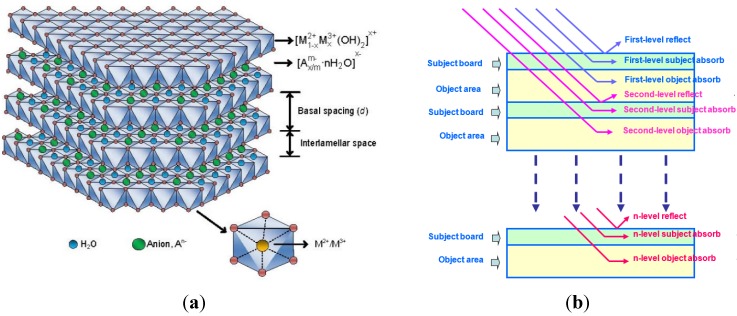
The structure (**a**) and mechanism of LDHs to impede the UV light (**b**).

Layered double hydroxides (LDH) have attracted considerable attention as an anti-UV agent in recent years. They belong to the class of anionic-layered materials formed by interlayer anions and laminates with a positive charge. As shown in Figure 1, LDHs have a multi-nested, layered structure. The inorganic layer sheets can function as a shield to physically impede the UV light; metal atoms of layer sheets and negative ions between layer sheets can chemically absorb the UV light [10,11,12,13,14]. Due to its fundamental structure, LDHs have the potential to improve the UV-aging resistance of bitumen. Xu and Wang’s research focused on the influence of different types of the LDHs on the aging properties of bitumen [15,16]. However, there is no research on the optical properties of LDH-modified bitumen which can give a fundamental explanation on the effect of LDHs on the aging properties of bitumen.

In this study, LDHs were used to modify the bitumen. The optical and UV-aging properties of modified-bitumen film were characterized. The results were expected to describe the mechanisms of aging resistance of LDH-modified bitumen. 

## 2. Materials and Test Methods

### 2.1. Materials and Preparation

**Figure 2:**
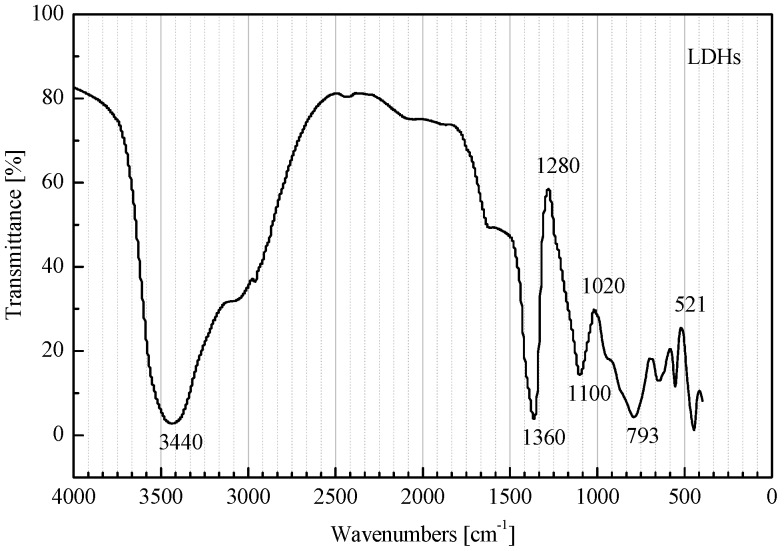
FTIR spectra of LDHs.

Base bitumen was provided by SK Corporation (Ulsan, Korea), with a penetration value of 89 dmm at 25 °C, a softening point of 45.5 °C and a dynamic viscosity of 330 Pa·s at 60 °C. LDHs were provided by RuiFa Chemical Company Ltd., (Jiangyin, China). It was a white powder, with a bulk density of 0.45 g/cm^3^ and a BET surface area of 27.7 m^2^/g. It contained Mg-Al layered double hydroxides, with the molecular formula as follows:

Mg_1−*x*_Al*_x_*(OH)_2_(CO_3_)*_x_*_/2_·*m*H_2_O

where *x* is the content variable of metallic elements, with 0.2 ≤ *x* ≤ 0.33; m is the amount of crystal water, with 0 ≤ *m* ≤ 2. Figure 2 shows the FTIR spectra of LDHs.

To prepare the LDH-modified bitumen sample, the base bitumen was first heated to be fluid at around 140 °C. Then, LDH powders (3 wt % and 5 wt % in this study) were slowly added to the base bitumen and sheared for 1 h by using a high-speed shearing mixer at a shearing speed of 4000 rpm, at 140 °C. The base bitumen also underwent the same shearing process to be a reference. On the surface of aggregate in the asphalt mixture, the film thickness of bitumen was several micro-meters. Therefore, the optical properties of bitumen film was determined at a micro-meter level. The film sample was prepared as follows: a certain amount of bitumen was dropped on a glass substrate; by warming the substrate on a heating plate, the bitumen spread freely by the effect of surface energy; by calculating the weight and the area of bitumen sample on the substrate, the thickness of the film was determined. 

### 2.2. Morphology and Optical Test

Fluorescence microscopy (FM) YS-100 (Nikon Company, Tokyo, Japan) was used to observe the morphology of LDHs in the bitumen film. Ultraviolet-Visible (UV-Vis) spectroscopy Lambda 750S, (PerkinElmer Company, Waltham, MA, USA) was adopted to characterize the absorbance, reflectance and transmittance of base and modified bitumen films. Polytetrafluoroethylene was used as a standard in the UV-Vis experiment. The wavelength range was selected within the UV region of 200–800 nm.

### 2.3. UV-Aging Properties

First, the thin film oven test (TFOT) was performed according to ASTM D 1754 to simulate the short-term aging of bitumen during the mixing process of the asphalt mixture [17]. Next, UV photo-oxidation test were used to simulate the long-term aging during the service life of bitumen in the asphalt pavement in an UV aging oven with the testing temperature of 50 °C and the UV strength of 10,000 μW/cm^2^ for six days. The thickness of film sample was 1250 μm.

Brookfield rotational viscometer (DV-II+Pro, Middleboro, MA, USA) was used to test the viscosity of the samples. The viscosity aging index (VAI) was calculated by Equation (1) and used to evaluate the aging degree of bitumen.

VAI = (*V*_2_ − *V*_1_)/*V*_1_ × 100% (1)

where *V*_1_ and *V*_2_ are viscosities of bitumen before and after UV aging at 60 °C, respectively.

The penetration retention rate (PRR) that is calculated by Equation (2) is often used to evaluate the aging degree of bitumen.

PRR = *P*_2_/*P*_1_ × 100% (2)

where *P*_1_ and *P*_2_ are the penetration of bitumen before and after UV aging, respectively.

The softening point increment (SPI) that is calculated by Equation (3) is often used to evaluate the aging degree of bitumen.

SPI = *SP*_2_−*SP*_1_ (3)

where *SP*_1_ and *SP*_2_ are the softening points of bitumen before and after UV aging, respectively.

To evaluate the physical properties of bitumen samples before and after aging, penetration (25 °C) and softening point (ring and ball method), were investigated according to the standard ASTM D5 [18] and ASTM D36 [19], respectively.

The Fourier Transform Infrared (FTIR) spectroscopy (Nexus, ThermoNicolet Corp., Waltham, MA, USA) is a technique used to identify functional groups in organic compounds at the molecular level. Infrared (IR) spectra can indicate the existence or absence of chemical functional groups. During the aging process of bitumen, the changes of carbonyl (at 1700 cm^−1^) and sulphoxide (at 1030 cm^−1^) are frequently used. In this study, we chose the area of the spectra bands between 600 cm^−1^ and 2000 cm^−1^ as the reference area [20]. For quantitative analysis, two structural indexes are calculated based on the band area according to Equations (4) and (5).

*I*_C=O_ = *A*_1700cm_^−1^/Σ*A* (4)

*I*_S=O_ = *A*_1030cm_^−1^/Σ*A* (5)

where *A*_1700cm_^−1^ is the area of the carbonyl band centered on 1700 cm^−1^ (calculated from 1650 to 1750 cm^−1^), *A*_1030cm_^−1^ is the area of the sulphoxide band centered on 1030 cm^−1^ (calculated from 980 to 1080 cm^−1^), and Σ*A* is the area of the spectra bands between 600 cm^−1^ and 2000 cm^−1^.

## 3. Results and Discussions

### 3.1. Morphology of Bitumen Film

**Figure 3:**
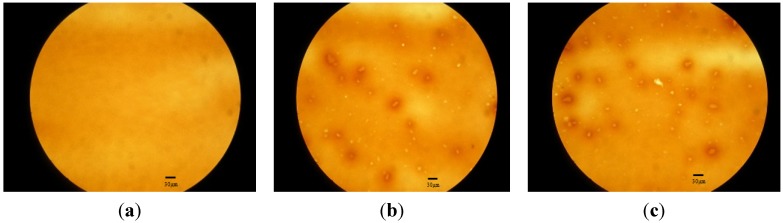
Morphology of base and modified bitumen films: (**a**) base; (**b**) with 3% LDHs; (**c**) with 5% LDHs, by using fluorescence microscopy, with 100 times magnification.

Figure 3 shows the dispersion of LDHs in the bitumen, by using fluorescence microscopy. As indicated, LDHs (white spots) are evenly dispersed in the bitumen (see Figure 3b,c).

### 3.2. Optical Properties of Base Bitumen

**Figure 4:**
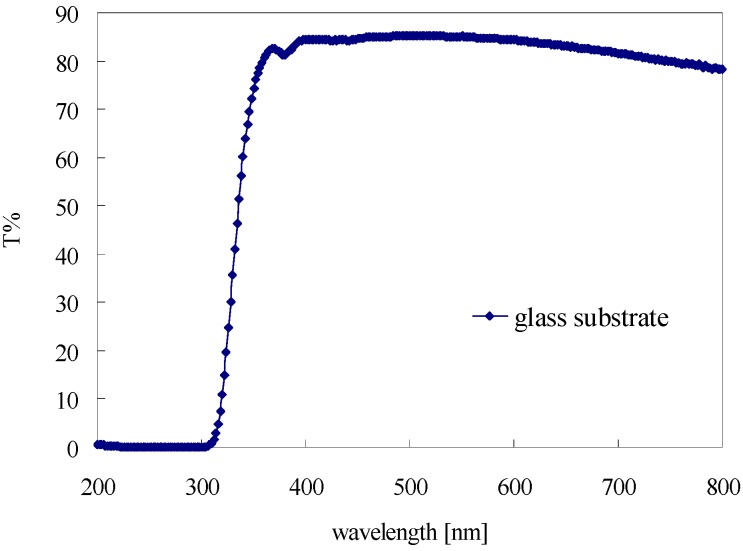
Transmittance of the glass substrate within the wavelength range between 200 and 800 nm.

The transmittance of the glass substrate within the wavelength range between 200 nm and 800 nm is shown in Figure 4. Within the wavelength range of 200–320 nm, the transmittance of the glass substrate is nearly zero due to the blocking effect of the particles in the glass. In the range of 300–800 nm, the transmittance of the glass substrate is higher than 80%. This means that most light in this range can transmit through the substrate. Therefore, the substrate has less influence on the transmittance of bitumen film above the wavelength of 300 nm. 

**Figure 5:**
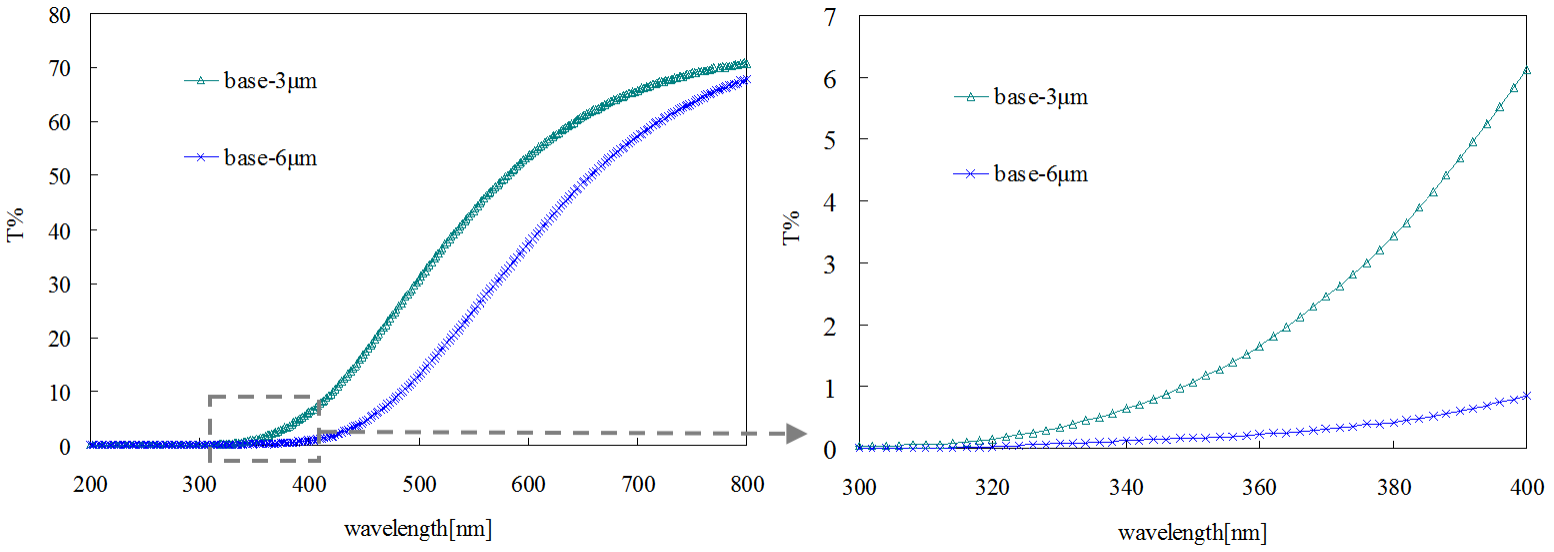
Transmittance of bitumen films with different thickness.

Two film samples of base bitumen with the thicknesses of 3 and 6 μm were prepared, and their transmittance curves are shown in Figure 5. In the range of 200–320 nm, the transmittance for both of them is nearly zero due to the blocking effect of the glass substrate. In the range of 320–400 nm, the transmittance of 6 μm-thick film is less than 1%, and the transmittance of 3 μm-thick film is much higher. This means that the thickness can influence highly the optical properties of bitumen film. In order to evaluate the effect of the LDHs on the optical properties of the base bitumen, a thickness of 3 μm for the film samples was selected.

### 3.3. Transmittance of Modified Bitumen Film

**Figure 6:**
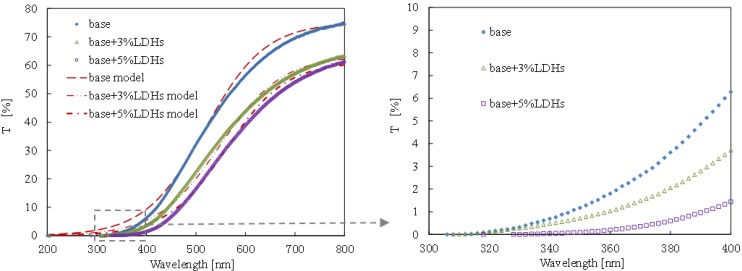
Transmittance of modified bitumen films with different LDH content.

Figure 6 shows the transmittance of base and modified bitumen films with a thickness of 3 μm. The transmittance of bitumen film decreased with the increase of LDH content, and 5% LDH-modified bitumen had the lowest transmittance, especially in the range of UV-A wavelength (320–400 nm). 

The S-model was used to describe the transmittance of bitumen films, as followed:

(6) $$T_{l}=\frac{M}{1+e\left[\frac{-\ln(81)}{\text{Δ}l}(l-l_{m})\right]}$$

where *M* is the maximum transmittance; Δ*l* is the interval of wavelength during which the growth of transmittance progresses from 10% to 90% of *M*; and *l_m_* is the wavelength at which the transmittance is half of *M*.

As shown in Figure 6, the data fit this model very well. As indicated in Table 1, *M* value decreased by 15% and 18%, respectively, due to the addition of 3% and 5% LDHs. The reason was that the LDHs had an enormous surface area (27.7 m^2^/g), and it can block the transmittance of light. 

**Table 1:** Parameters of S-model for base and modified bitumens.

Parameters	Base	Base + 3% LDHs	Base + 5% LDHs
*M* (%)	74.45	63.32	61.22
Δ *l* (nm)	266	276	264
*l_m_* (nm)	518	548	561
*R*^2^	0.9980	0.9974	0.9979

Within the wavelength range of 300–400 nm for the UV-light (see the bottom of Figure 6), the LDHs decreased the transmittance more. For example, the transmittance of base bitumen at 400 nm was 6.3%, and that for bitumen with 3% LDHs, it was reduced by 44.4%. With respect to bitumen with 5% LDHs, the transmittance was reduced by 76%. The fact indicated is that the LDHs had higher efficiency in reducing the light transmittance at the UV-light range than at the visible-light range. The reason was that the LDHs had a multi-nested layered structure (see Figure 1) which can effectively impede the UV light, and meanwhile the metal atoms of layer sheets and negative ions between layer sheets can chemically absorb the UV light. This means that the addition of LDHs into bitumen can improve the UV-aging resistance. 

### 3.4. Absorbance of Modified Bitumen Film

**Figure 7:**
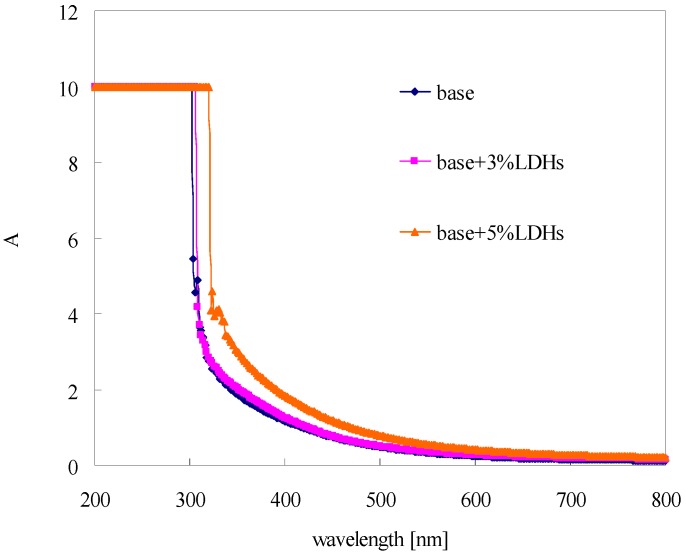
Absorbance of modified bitumen film with different content of LDHs.

Figure 7 shows the absorbance of modified bitumen film with different content of LDHs. In the range of 200–320 nm, the absorbance for all film samples is quite high. It indicated that bitumen (with and without LDHs) has high absorbance in the range of UV-B (290–320 nm) and UV-C (220–290 nm). However, in the field, UV-B and UV-C from sunshine hardly reach the surface of the Earth. Therefore, their aging effect on the bitumen can be ignored. In the range of UV-A (320–400 nm), the bitumen with 3% LDHs exhibited the similar absorbance to base bitumen. While the absorbance of 5% LDH-modified bitumen was higher due to the absorbance capacity of LDHs. As described in Figure 1, LDHs can absorb the photo-energy from the UV light and convert it into heat energy, and finally dissipate into the environment. In this way, the bitumen was protected from the penetration of the UV light.

### 3.5. Reflectance of Modified Bitumen

**Figure 8:**
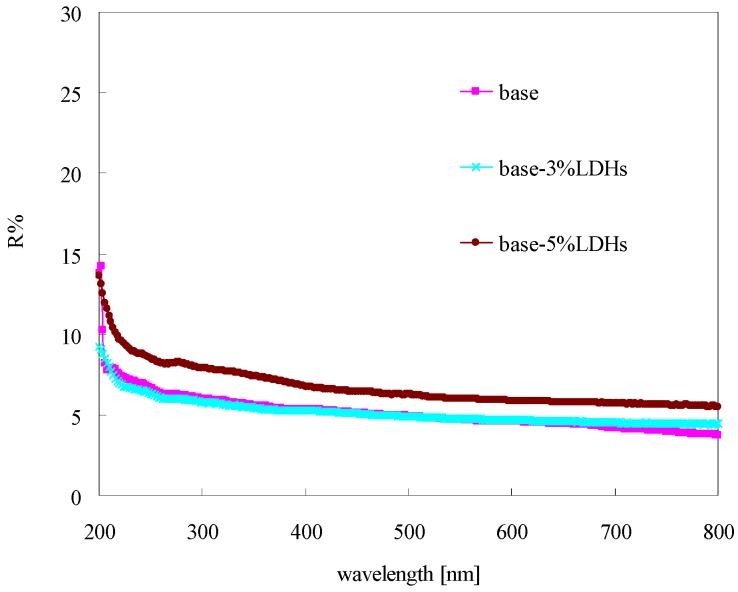
Reflectance of modified bitumen film with different content of LDHs.

Figure 8 shows the reflectance result for the base and LDH-modified bitumens. As indicated, the reflectance of bitumen films decreased with the increase of wavelength, and became stable when the wavelength was larger than 400 nm (*i.e.*, at the visible-light range). The bitumen with 5% LDHs had a higher reflectance at the UV-light range (200–400 nm). However, the bitumen with 3% LDHs had a comparable reflectance with the base bitumen. Therefore, a certain amount of LDHs (here 5%) can increase the reflectance of bitumen to the UV-light. 

### 3.6. UV-Aging Properties

#### 3.6.1. Rheological Properties

The viscosities of base and modified bitumen before and after UV aging are given in Table 2. As indicated, the addition of LDHs increased the viscosity of base bitumen. After the UV aging, the viscosity of each bitumen also increased. However, the VAI value for LDH-modified bitumen was lower than the base bitumen. Moreover, the greater the content of LDHs, the lower the VAI value. 5 wt % LDH-modified bitumen had the lowest VAI value, and exhibited the best performance of UV aging resistance. 

**Table 2:** Viscosity of bitumen before and after UV aging at 60 °C.

LDHs Content (wt %)	*V*_1_ of Fresh Bitumen (Pa·s)	*V*_2_ of Bitumen After UV Aging (Pa·s)	VAI (%)
0	330	660	50
3	500	720	44
5	550	735	33.6

**Figure 9:**
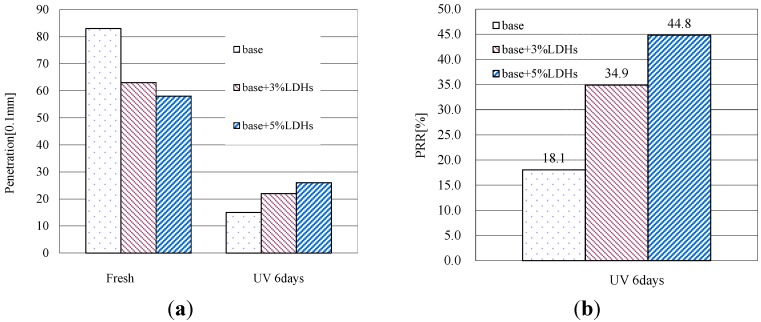
(**a**) Penetration of bitumen before and after UV aging; (**b**) PRR of bitumen after UV aging.

Figure 9a shows the penetration of base bitumen and LDHs modified bitumen before and after UV aging. As indicated, the addition of LDHs decreased the penetration value of base bitumen because the LDHs made the bitumen more viscous. However, this value for the base bitumen became lower than that for modified bitumen after UV aging. As shown in Figure 9b, the base bitumen had the lowest PRR of 18.1%, meaning poor UV-aging resistance. After the addition with 5 wt % of LDHs, this value increased to 44.8%. This indicated that the addition of LDHs can improve the UV-aging resistance of base bitumen. 

**Figure 10:**
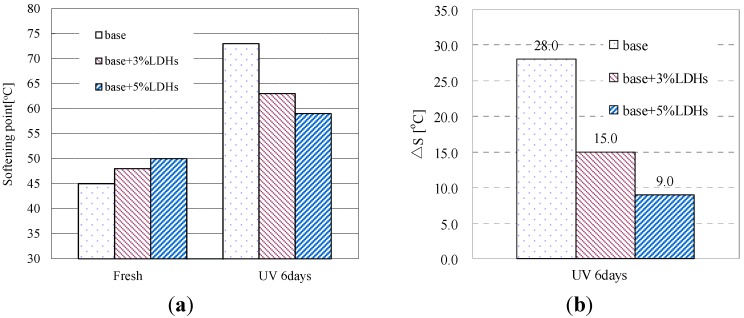
(**a**) Softening point of bitumen before and after UV aging; (**b**) SPI of bitumen after UV aging.

Figure 10a shows the softening points of base bitumen and LDH-modified bitumen before and after UV aging. As indicated, the addition of LDHs increased the softening point value of base bitumen because the LDHs made the bitumen more viscous. However, this value for the base bitumen became higher than that for modified bitumen after UV aging. As shown in Figure 10b, the base bitumen had the highest SPI of 28 °C, meaning poor UV-aging resistance. After mixed with 5 wt % of LDHs, this value decreased to 9.0 °C. This indicated that the addition of LDHs can improve the UV-aging resistance of base bitumen. This corresponded well with the results of viscosity and penetration.

#### 3.6.2. Chemical Properties

**Figure 11:**
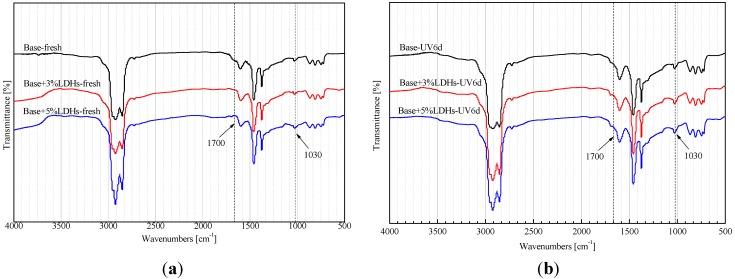
FTIR spectra of base and LDH-modified bitumens (**a**) before and (**b**) after UV-aging.

Figure 11 shows the FTIR spectra of base and LDH-modified bitumens before and after UV-aging. The UV-aging process can accelerate the oxidation of bitumens, and increase the peak area of carbonyl at 1700 cm^−1^ and sulphoxide at 1030 cm^−1^. The structure-change indexes of base and modified bitumens are given in Table 3. After UV aging, the carbonyl index (*I*_C=O_) of base bitumen increased by 0.0184, and the sulphoxide index (*I*_S=O_) by 0.0370. However, the *I*_C=O_ values of 3% and 5% LDH-modified bitumen only increased by 0.0163 and 0.0123, and the *I*_S=O_ by 0.0290 and 0.0200. Therefore, the addition of LDHs can inhibit the oxidation of bitumen during UV aging.

**Table 3:** Structure-change indexes of base and modified bitumen before and after UV aging.

Sample	Index	Fresh	UV Aging
base	*I*_C=O_	0.0036	0.0220
	*I*_S=O_	0.0100	0.0480
Base + 3% LDHs	*I*_C=O_	0.0036	0.0199
	*I*_S=O_	0.0100	0.0396
Base + 5% LDHs	*I*_C=O_	0.0037	0.0160
	*I*_S=O_	0.0100	0.0300

## 4. Conclusions

LDH-modified bitumen was prepared by the melt-blending method. According to the test results, LDHs had a significant influence on optical and UV-aging properties of base bitumen, and some conclusions can be drawn as follows:

Layered double hydroxides (LDHs) had two functions in the modification of base bitumen: one was that LDHs can effectively absorb the UV light and convert it into heat energy; another one was that LDHs can reflect part of UV light. Based on these two reasons, LDHs can improve the UV-aging resistance of bitumen, especially with 5% content of LDHs. This implied that the addition of LDHs into bitumen had the potential to prolong the service life of asphalt pavement. 
